# Numerical Simulation Research on Partial Discharge of Particle Defects at Epoxy Interface Excited by High-Frequency Sinusoidal Voltage

**DOI:** 10.3390/polym15102320

**Published:** 2023-05-16

**Authors:** Chen Chen, Jian Wang, Jingrui Wang, Zhihui Li, Rui Guo, Hu Jin, Botao Li

**Affiliations:** 1 State Key Laboratory of Alternate Electrical Power System with Renewable Energy Sources, North China Electric Power University, Beijing 102206, China; chenchen921197@163.com (C.C.); wang_jingrui@ncepu.edu.cn (J.W.); lzh_bryant@ncepu.edu.cn (Z.L.); 2 Heze Power Supply Company, Heze 274000, China; 15617622745@163.com; 3 Electric Power Research Institute, CSG, Guangzhou 510630, China; jinhu124@163.com; 4 Rizhao Power Supply Company, Rizhao 276800, China; qingxinjue123@163.com

**Keywords:** partial discharge simulation, high-frequency sinusoidal voltage, particle defect, surface damage, gas–solid interface

## Abstract

In order to improve the effectiveness of partial discharge detection in attached metal particle insulators, this paper proposes a partial discharge detection method for particle defects in insulators under high-frequency sinusoidal voltage excitation. In order to study the development process of partial discharge under high-frequency electrical stress, a two-dimensional plasma simulation model of partial discharge with particle defects at the epoxy interface is established under plate–plate electrode structure, which realizes the dynamic simulation of particulate defect partial discharge. By studying the microscopic mechanism of partial discharge, the spatial and temporal distribution characteristics of microscopic parameters such as electron density, electron temperature, and surface charge density are obtained. Based on this simulation model, this paper further studies the partial discharge characteristics of epoxy interface particle defects at different frequencies, and verifies the accuracy of the model from two aspects of discharge intensity and surface damages through experimental means. The results show that with the increase in the frequency of applied voltage, the amplitude of electron temperature shows an increasing trend. However, the surface charge density gradually decreases with the increase in frequency. These two factors make partial discharge severest when the frequency of the applied voltage is 15 kHz.

## 1. Introduction

With the rapid development of Ultra-High Voltage transmission technology, gas insulated metal enclosed transmission line (GIL) has attracted wide attention and is characterized by a large capacity, high voltage, and good electromagnetic compatibility [1,2,3]. Judging from the operating experience of GIL in recent decades, insulation failure has always been one of the most important factors affecting the safety and reliability of GIL. In particular, metal particles generated during production, transportation, installation, and operation of GIL equipment are the most common insulation defects [4]. If these insulation defects cannot be found and dealt with in time, it is likely to endanger other non-faulty components and even develop into insulation faults, threatening the safety of the power supply. Therefore, improving the efficiency of particle defect detection and identification is essential to guarantee the safe operation of GIL.

The characteristics of partial discharge are related to different excitation conditions, different insulating materials, and different types of discharge [5]. At present, the research on partial discharge characteristics and defect diagnosis has mainly carried out by means of standard lightning impulse voltage and oscillatory lightning impulse voltage [6,7,8]. Compared with traditional power frequency voltages, the partial discharge excited by lightning impulse voltage is more intense. However, the interference signal generated by a lightning impulse voltage generator has greatly influences the validity of the partial discharge measurement results. This makes the detection of partial discharge defects not fully applicable to the needs of engineering. The literature [9] shows that the characteristics of partial discharge are related to the frequency of the applied voltage. Li Qingmin used high-frequency sinusoidal voltage to study the influence of frequency on the partial discharge of polyimide and drew the conclusion that, under the same external conditions, the characteristic parameters of power frequency partial discharge (average discharge, maximum discharge, discharge times, and discharge energy) are far less than those under high-frequency sinusoidal voltage. Based on this, it is of great academic and engineering significance to study the partial discharge characteristics and diagnosis methods of GIL internal insulation defects under high-frequency sinusoidal voltage.

Experiments and operation experience show that the deterioration of GIL insulation strength caused by surface flashover of insulators attached with metal particles is severer than that caused by air gap breakdown [10]. Based on this, this paper mainly studies creeping discharge characteristics and diagnosis methods of insulators with particle defects. The current research on insulators attached with metal particles mainly focuses on the following aspects: (1) The distortion of electric field on surface of insulator with particle defects. For example, the literature [11,12] established a three-dimensional simulation model of the basin insulator attached with metal particles and calculated the electric field intensity distribution on the surface of the basin insulator. (2) The influence of particle defects on the surface charge accumulation of insulators. For example, the literature [13] optimized the design of the capacitance probe and explored in detail the distribution and accumulation of surface charge of the insulator; considering particle behaviors such as generation and diffusion of gas side charge, the charge accumulation process on the insulator surface is analyzed in detail in the literature [14]. (3) Research on flashover characteristics of insulators induced by metal particles. For example, the literature [15] explored the detection and development law of surface flashover of insulators with metal particles based on actual insulators. The literature [16] shows that the surface flashover mechanism of insulators attached with metal particles is related to surface electric field distribution and surface charge accumulation. The literature [17] investigated the characteristics of dielectric blocking glow discharge under different discharge waveforms and concluded that the root mean square value of the current is greatest under square waves. Although the above researches have made some progress, the current research on the discharge characteristics of insulators attached with metal particles mainly involves the analysis of flashover characteristics. However, the research on partial discharge characteristics is mainly conducted through experimental means, which cannot completely clarify the influence mechanism of partial discharge in insulators with particle defects and how to improve the validity of the detection results of particle defects. Therefore, it is necessary to study the micro mechanism of particle defect partial discharge of epoxy insulator under high-frequency sinusoidal voltage excitation.

Based on this, this paper proposes a partial discharge detection method for particle defects of epoxy insulator under high-frequency sinusoidal voltage excitation. For epoxy insulators attached with metal particles, a two-dimensional plasma simulation model for the partial discharge of epoxy interface particle defects under the plate–plate electrode structure was built to realize the dynamic simulation of the development process of particulate defect partial discharge. The research results in this paper provide a method for the quantitative analysis of particle defects at the epoxy interface and provide effective guidance for the detection of partial discharge of epoxy insulators attached with particles.

## 2. Plasma Simulation Model for Partial Discharge of Metal Particles at Epoxy Interface

The numerical simulation of partial discharge is very complicated. Both the transport characteristics of charged particles and the chemical reactions between plasmas need to be considered during the discharge. Traditional partial discharge numerical models include fluid models and particle models. Although they have their own advantages, in general, there are still certain shortcomings. Based on this, this paper builds a fluid–chemical hybrid numerical model by coupling the fluid model and particle model. In addition, in order to reduce the difficulty of solving the model, the complex chemical reactions are reasonably simplified by using reaction sets. Based on this, the particle transport equation, plasma chemical reaction, boundary conditions, and construction of the geometric model are described in detail.

### 2.1. Particle Transport Equation

In order to elaborate on the microscopic mechanism of the particle defects’ partial discharge at the epoxy interface, the fluid–chemical hybrid model couples the electronic continuity equation, the multi-component diffusion and transport equations of heavy particles, the energy equation, and the Poisson equation to describe the collision, ionization, attachment, and recombination process of charged particles during the discharge, so as to achieve an accurate description of the generation, diffusion, migration, and disappearance of charged particles. In addition, the photoionization of the epoxy interface and the emission of secondary electrons need to be considered.

The electron continuity equation is usually described via the Boltzmann equation, which is difficult to solve effectively. When ignoring the electronic convection caused by the external electric field, the electronic continuity equation is shown in Formulas (1) and (2) [18]:

(1) $$\frac{\partial }{\partial t}(n_{e})+\nabla \cdot \Gamma_{e}=R_{e}+S_{e}$$

(2) $$\Gamma_{e}=-(\mu_{e}E)n_{e}-\nabla (D_{e}n_{e})$$

In the formula, *n_e_* is the electron density; ***Γ_e_*** is the electron flux; *R_e_* is the electron source term determined by the plasma reaction and the surface reaction of the insulating medium; *S_e_* is the photoionization coefficient. The calculation formula is shown in Formula (3); *μ_e_* is the electron mobility; ***D_e_*** is the electron diffusion coefficient.

(3) $$S_{e}=\frac{1}{4\pi }\cdot \frac{P_{q}}{P+P_{q}}\times \int_{V}d^{3}r_{1}\frac{S_{ion}(r_{1})}{|r-r_{1}{|}^{2}}\Psi (|r-r_{1}|\cdot P)$$

In the formula, *P* is atmospheric pressure; *P_q_* is the gas pressure of the excited atom; *Ψ*(|*r* − *r*_1_|·*P*) is the absorption coefficient of ionizing radiation; *r* is the distance between photon radiation and absorption.

In order to elaborate on the mechanism of electron energy generation and loss during partial discharge, the electron energy constraint equation is shown in Formulas (4) and (5):

(4) $$\frac{\partial }{\partial t}(n_{\varepsilon })+\nabla \cdot \Gamma_{\varepsilon }+E\cdot \Gamma_{e}=S_{en}$$

(5) $$\Gamma_{\varepsilon }=-(\mu_{\varepsilon }E)n_{\varepsilon }-\nabla (D_{\varepsilon }n_{\varepsilon })$$

In the formula, *n_ε_* is the electron energy density; ***Γ_ε_*** is the electron energy flux; *S_en_* is the energy lost or gained during the inelastic collision between electrons and heavy particles; *μ_ε_* is the electron energy mobility; ***D_ε_*** is the diffusion coefficient of electron energy.

*R_e_* in Formula (1) and *S_en_* in Formula (4) are determined by plasma chemical reactions. When the reaction rate is determined, the calculation formulas of *R_e_* and *S_en_* are shown in Formulas (6) and (7):

(6) $$R_{e}=\sum_{g=1}^{M_{1}}x_{g}k_{g}N_{n}n_{e}+\sum_{h=1}^{M_{2}}x_{h}\alpha_{h}N_{n}|\Gamma_{e}|$$

(7) $$S_{en}=\sum_{g=1}^{P_{1}}x_{g}k_{g}N_{n}n_{e}\Delta \varepsilon_{g}+\sum_{h=1}^{P_{2}}x_{h}\alpha_{h}N_{n}|\Gamma_{e}|\Delta \varepsilon_{h}$$

In the formula, *x_g_* and *x_h_* are the respective mole fractions of reaction *g* and *h*; *k_g_* is the reaction rate coefficient of reaction *g*; *α_h_* is the Townsend coefficient of response *h*; *N_n_* is the density of heavy particles; Δ*ε_g_* and Δ*ε_h_* are the energy loss of reaction *g* and *h*, respectively.

In the process of partial discharge at the epoxy interface, the multi-component diffusion and transport equations of heavy particles is shown in Formula (8):

(8) $$\rho \frac{\partial }{\partial t}(w_{k})+\rho (u\cdot \nabla )w_{k}=\nabla \cdot j_{k}+R_{k}+S_{e}$$

In the formula, *ρ* is density of the mixture; *w_k_* is the mass fraction of substance *k*; ***u*** is the average fluid velocity; *j_k_* is the diffusion flux; *R_k_* is the change rate of substance *k*.

The electric field is calculated by the Poisson equation as shown in Formulas (9) and (10):

(9) $$-\nabla \cdot \varepsilon_{0}\varepsilon_{r}\nabla V=\rho_{v}$$

(10) $$\rho_{v}=q(\sum_{k=1}^{N}Z_{k}n_{k}-n_{e})$$

In the formula, *V* is the space electromotive force; *ρ_v_* is the space charge density; *q* is the elementary charge; *Z_k_* is the amount of charge carried by substance *k*; *n_k_* is the density of substance *k*.

### 2.2. Plasma Chemical Reaction

In the process of partial discharge, air is a mixed gas involving dozens of particles and hundreds of chemical reactions, and its chemical reaction system is relatively difficult to establish. These things considered, this paper mainly studies the partial discharge characteristics of epoxy interface particle defects under high-frequency sinusoidal voltage excitation, while the specific conditions of some reactions are not addressed. Moreover, according to the theory of fluid dynamics, the transport equations of various particles can be described by the convection–diffusion equations of positive ions, negative ions, and neutral particles. Therefore, this paper uses the reaction set to reasonably simplify the complex chemical reaction to reduce the difficulty of solving the model.

The plasma chemical reaction system considered in this paper is shown in Table 1. The air component is simplified reasonably, and the chemical reaction of N_2_ and O_2_ is no longer treated separately but replaced by substance A. Substance A can either ionize to generate positive ions (p) or attach electrons to generate negative ions (n).

### 2.3. Boundary Condition Setting and Geometric Modeling

The effect of epoxy interface on partial discharge is mainly reflected in two aspects. (1) The charged particles collide with the epoxy interface, causing surface reactions and generating secondary electrons; (2) Surface charge accumulates at the epoxy interface, changing the electric field distribution at the epoxy interface and promoting the development of partial discharge.

During partial discharge, the changes in electron density and electron energy at the epoxy interface are related to thermal motion, electromigration, and secondary electron emission. Based on this, the wall boundary conditions are used to describe the thermal motion, electromigration, and secondary electron emission process when the charged particles are in contact with the epoxy interface during the partial discharge. The flux boundaries of electron density and electron energy density are shown in Formulas (11) and (12):

(11) $$n\cdot \Gamma_{e}=\frac{1}{2}v_{e,th}n_{e}+\alpha_{e}n\cdot (\mu_{e}\cdot E)n_{e}-\sum \gamma_{e}(\Gamma \cdot n)$$

(12) $$n\cdot \Gamma_{\varepsilon }=\frac{5}{6}v_{e,th}n_{\varepsilon }+\alpha_{e}n\cdot (\mu_{\varepsilon }\cdot E)n_{\varepsilon }-\sum \varepsilon \gamma_{e}(\Gamma \cdot n)$$

In the formula, ***n*** is the normal vector of the gas gap pointing to between the electrode and the epoxy interface; The first, second, and third terms on the right side of the formula represent the thermal motion, electromigration, and secondary electron emission of electrons, respectively; *V*_*e*,*th*_ is the velocity of electron thermal movement; The value of *α_e_* depends on the direction of the electric field. When the direction of the electric field and the boundary normal vector are the same, the value of *α_e_* is 0. Otherwise, *α_e_* is equal to 1; *γ_e_* is the secondary electron emission coefficient. According to the literature [19], the secondary electron emission coefficient of the electrode surface is set to 0.004, and the secondary electron emission coefficient of the epoxy interface is set to 0.5; *ε* is the average energy of secondary electrons, and it is set to 2.5 eV.

In the process of partial discharge, the surface charge accumulated at the epoxy interface causes discontinuity in the electric displacement vector on both sides of the wall boundary. The boundary conditions for charge accumulation at the epoxy interface are shown in Formula (13):

(13) $$n\cdot (D_{1}-D_{2})=\sigma_{s}$$

In the formula, ***D*_1_** and ***D*_2_** are the electric displacement vectors on both sides of the wall boundary; *σ_s_* is the surface charge density. The calculation formula is shown in Formula (14).

(14) $$\frac{\partial \sigma_{s}}{\partial t}=n\cdot J_{i}+n\cdot J_{e}$$

In the formula, ***n·J_i_*** is the normal component of the total ion density at the epoxy interface; ***n·J_e_*** is the normal component of the total electron density at the epoxy interface.

### 2.4. Geometric Model

**Figure 1:**
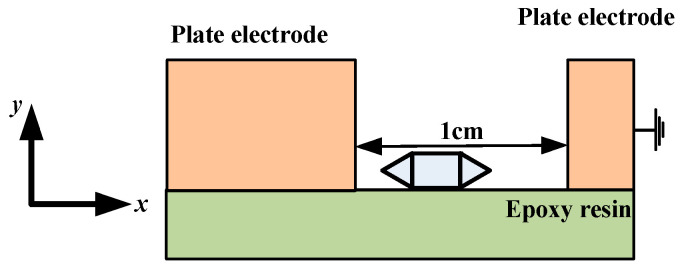
Simplified model of the geometric structure.

Particle defects at the epoxy interface can induce surface charge accumulation and lead to surface electric field distortion, which causes partial discharge. In addition, the operating experience shows that linear metal particles suffer the most damage due to insulation. Based on this, a two-dimensional geometric model of linear metal particles under a flat plate electrode structure was built using the COMSOL software, and a plasma simulation model based on the aforementioned was established in the plasma module of the COMSOL software to describe the development process of partial discharge in particle defects at the epoxy interface. In order to reduce the complexity of modeling and the difficulty of solving the model, the geometric structure was simplified into a two-dimensional model, as shown in Figure 1. The model consisted of plate electrode (thickness = 0.6 cm), epoxy resin (thickness = 0.2 cm), and linear metal particles (length = 0.4 cm, thickness = 0.1 cm, radius of curvature = 5 cm). In addition, the distance between the two-plate electrodes was 1 cm.

## 3. Partial Discharge Evolution Characteristics of Metal Particle at Epoxy Interface

During partial discharge, insulators with particle defects will aggravate the decomposition of epoxy resin, leading to the degradation of epoxy insulators and even causing insulator surface flashover. However, the influence mechanism of metal particles on the discharge development in insulators is still unclear. Based on this, this paper carried out a numerical simulation study of the partial discharge of epoxy interface particle defects under the excitation of high-frequency sinusoidal voltage. In order to simulate the real experimental conditions as closely as possible, the ambient temperature was set to 20 °C; The gas pressure was set to 1.013 × 105 Pa; and the voltage was set to 10 kV, which was consistent with the voltage applied during the experiment. Through a large number of numerical calculations, the distribution rule of partial discharge of epoxy interface particle defects was explored. Furthermore, according to the distribution characteristics of electron density and electric field intensity during the partial discharge, the spatial and temporal distribution features of partial discharge parameters were obtained.

### 3.1. Temporal and Spatial Distribution Characteristics for Partial Discharge Characteristic Atlas of Metal Particle

**Figure 2:**
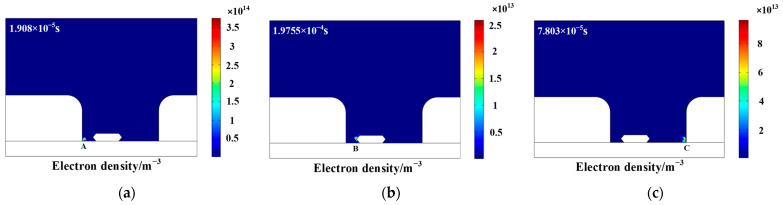
The numerical distribution chart of the electron density. (**a**) point A; (**b**) point B; (**c**) point C.

**Figure 3:**
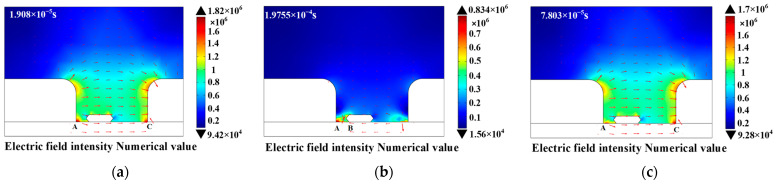
The numerical distribution chart of the electric field intensity. (**a**) point A; (**b**) point B; (**c**) point C.

**Figure 4:**
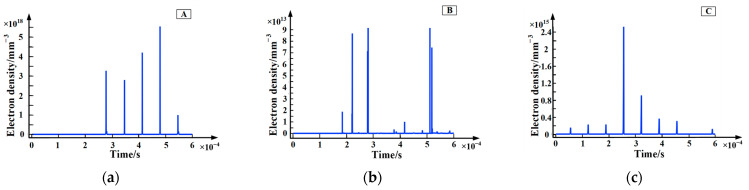
The chart of electron density with time at different positions. (**a**) point A; (**b**) point B; (**c**) point C.

In the process of partial discharge, the numerical distribution of electron density and electric field intensity in the discharge area can directly characterize the development process of partial discharge of epoxy interface particle defects, which is of great significance to clarify the influence mechanism of metal particles on partial discharge. In order to explore the temporal and spatial distribution of the partial discharge characteristic Atlas of metal particles, a high-frequency sinusoidal voltage of 10 kV and 10 kHz was used as an external voltage source to study the partial discharge characteristics of particle defects at the epoxy interface. Figure 2 and Figure 3 are the numerical distribution charts of the electron density and electric field intensity during the discharge process. The red arrows in Figure 3 represent the direction of the electric field intensity. It can be seen that the partial discharge is mainly concentrated at the three junctions of the electrode: the epoxy interface and the air (A, C) and the end of the metal particles near the electrode side (B). In addition, the time when the first corona discharge occurs at point A and point C is much earlier than that at point B. Furthermore, the electron density and electric field strength are obviously consistent. As the position of the corona discharge changes, the position of the maximum electric field intensity also changes. Finally, through calculation, the maximum value of the electric field strength can reach 10 kV/cm, which is basically consistent with the calculation results in the literature [20]. In order to further explore the severity of corona discharge at different locations, this paper studies the relationship between electron density at different locations and time, as shown in Figure 4. In view of the amplitude and distribution range of electron density, the electron density of point A is far higher than that of point C, and the electron density of point C is far higher than that of point B, which is basically consistent with the phenomenon observed in the simulation process. In other words, at the initial stage of partial discharge, the degree of partial discharge near point B is small, and it has little effect on the partial discharge at the three junction points (A, C). With the development of partial discharge, the degree of partial discharge at B gradually intensifies, making the degree of electric field distortion between point A and point B far higher than that between point C and point B, which results in partial discharge being severer at point A than that at point C.

### 3.2. Distribution Characteristics of Partial Discharge Current Density for Metal Particles

**Figure 5:**
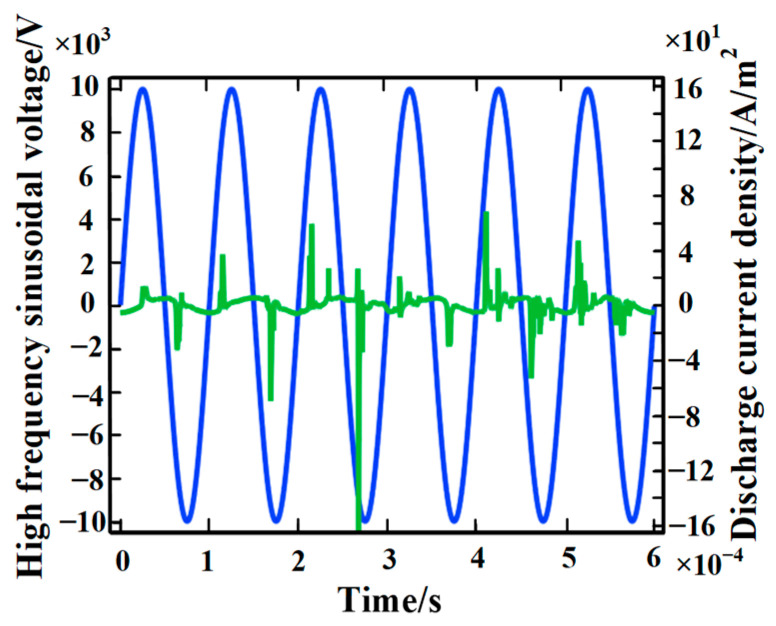
Chart of the partial discharge current density with time.

The discharge current can characterize the evolution rule of the partial discharge of epoxy interface particle defects. With the severest location of partial discharge being at point A, through a large number of simulation calculations, we obtained a chart of the partial discharge current density at point A with time, as shown in Figure 5. The blue curve is the applied voltage and the green curve is the partial discharge current density. It can be found from the figure that partial discharge has strong randomness, but the pulse discharge current is mainly concentrated near the peak of the high-frequency sinusoidal voltage. In addition, the partial discharge current is very small before the pulse discharge current is formed. The reason for this is that the greater the total electric field intensity in the discharge area, the greater the probability of partial discharge. The instantaneous voltage near the peak of the sinusoidal voltage is larger, so the total electric field intensity in the discharge area is relatively larger. Under a strong electric field, a large amount of collision ionization occurs near point A, producing more positive ions and electrons. Since electrons move faster than positive ions, it can be considered that positive ions almost stay in the ionization region. At the epoxy interface, the rapid reaction of positive ions (Section 2.2, Reaction 5) aggravates the collision ionization of electrons and promotes the development of partial discharge. With the development of partial discharge, the electric field formed by the space charge increases, which promotes the decrease of the total electric field intensity in the discharge area. Therefore, the electrons’ energy (obtained from the electric field) decreases, and the corona gradually disappears. The single partial discharge ends and the discharge current continues at a low value.

## 4. Partial Discharge Characteristics Analysis for Metal Particles of Epoxy Interface at Different Frequencies

During partial discharge, a large number of charged particles undergo collision, ionization, attachment, and recombination processes. The internal self-consistent field is constantly affected by chemical reactions between particles, so the microscopic mechanism of partial discharge is very complex. In order to understand the generation, diffusion, migration, and disappearance process of charged particles and fully reflect the microscopic mechanism of partial discharge, we took a high-frequency sinusoidal voltage of 10 kV as the applied voltage source and statistically analyzed the distribution rules of particle density, electron temperature, and surface charge density at point A at different frequencies through a large number of simulation calculations. In addition, the accuracy of the model was verified from two aspects of discharge form and discharge intensity by experimental means. In this paper, the optimal frequency of the partial discharge of epoxy interface particle defects excited by high-frequency sinusoidal voltage is determined, and the influence mechanism of frequency increase on partial discharge is revealed.

### 4.1. Particle Dynamics Behavior

**Figure 6:**
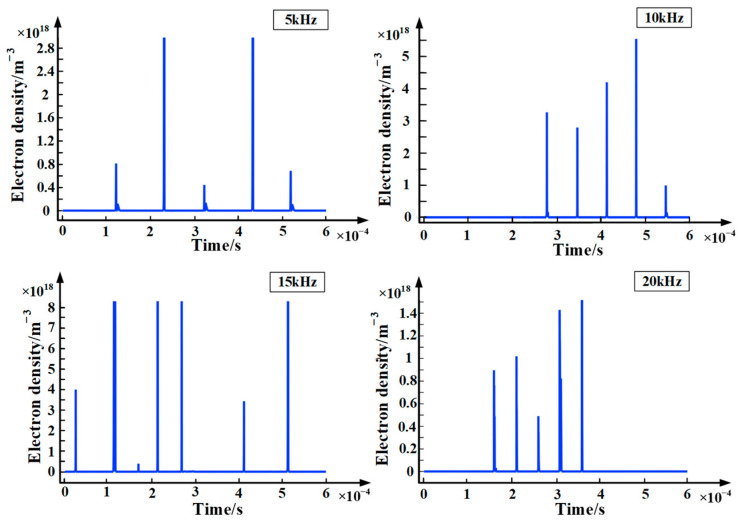
Chart of electron density with time at different frequencies.

Since the drift velocity of electrons is about 3–4 orders of magnitude higher than that of positive and negative ions, the partial discharge current is mainly caused by the drift motion of electrons. Based on this, this paper uses the electron density to characterize the severity of partial discharge. Figure 6 shows the chart of electron density with time at different frequencies. It can be seen from the figure that the variation of electron density is similar to the variation of partial discharge current density, both of which show a certain randomness. In addition, the electron density is maintained at about 10^18^ m^−3^ during the partial discharge. Furthermore, in view of the amplitude and distribution range of electron density, the electron density is largest when the applied voltage frequency is 15 kHz. In other words, the partial discharge of epoxy interface particle defects is severest when the frequency of the applied voltage is 15 kHz.

**Figure 7:**
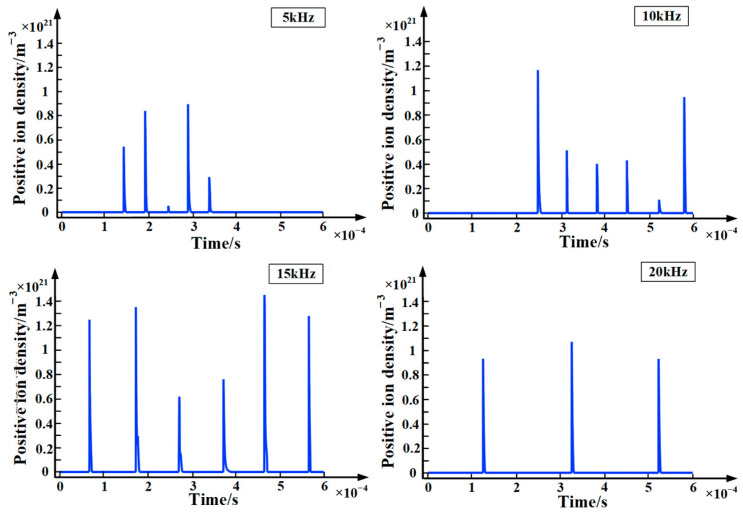
Chart of positive ions with time at different frequencies.

**Figure 8:**
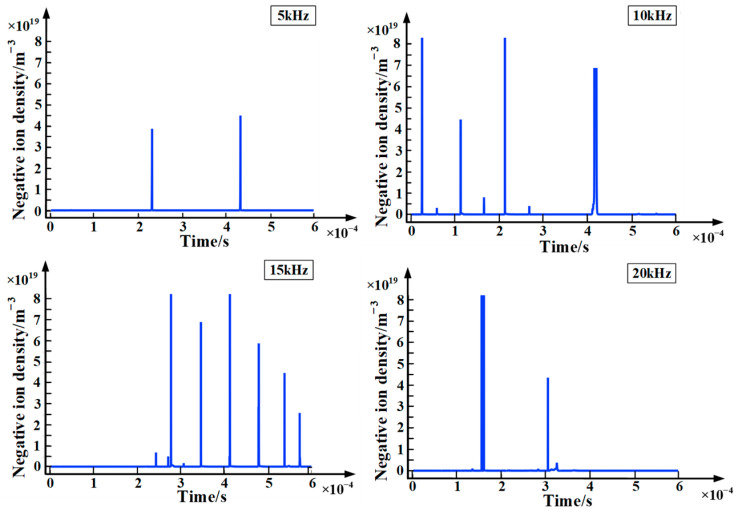
Chart of negative ions with time at different frequencies.

Figure 7 and Figure 8 are the charts of positive and negative ions with time at different frequencies. It can be seen from the figures that the variation of positive ions is similar to the variation of electron density. This is because both positive ions and electrons are generated by impact ionization. In addition, when the applied voltage frequency is 15 kHz, the density of positive and negative ions is the largest, which is consistent with the electron density. Furthermore, the magnitude of the positive ion density is about two orders of magnitude higher than the negative ion density. The reasons for this are as follows: (1) During partial discharge, collision ionization plays a leading role and generates a large number of positive ions. (2) The migration speed of electrons is relatively fast, and it is difficult for neutral particles to capture electrons to form negative ions in a strong electric field. (3) Negative ions are easy to react to generate neutral particles with surrounding positive ions. For the above reasons, the density of negative ions is lower than that of positive ions.

### 4.2. Electron Temperature Distribution Characteristics

**Figure 9:**
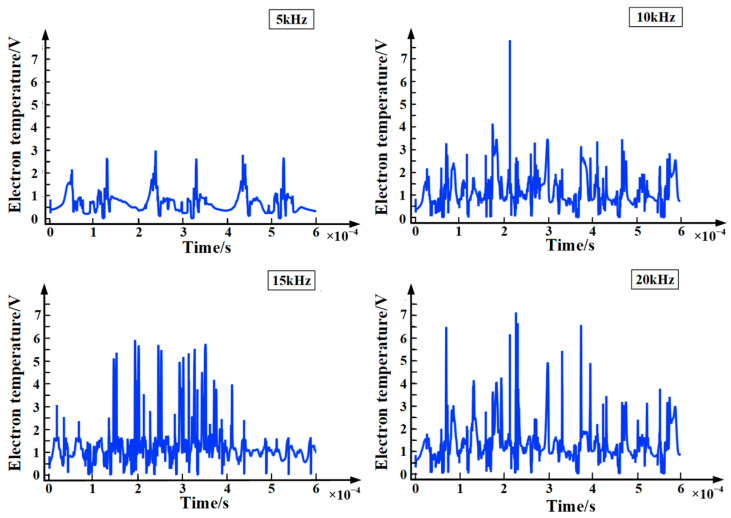
Chart of electron temperature with time at different frequencies.

Electron temperature is a characteristic of electron energy and the main medium of energy transfer in the process of partial discharge. Studying the relationship between variations in electron temperature and time can help to understand the factors affecting the severity of partial discharge and the rate of energy transfer in the process of partial discharge. Figure 9 shows the chart of electron temperature with time at different frequencies. It can be seen from the figure that, during the partial discharge, the electron temperature is maintained at about 1–7 eV, which is consistent with the results in the literature [21]. In addition, as the frequency of the applied voltage increases, the amplitude of the electron temperature gradually increases. In the initial stage of partial discharge, the electron energy near point A is relatively small. Under a strong electric field, electrons obtain energy through the Joule heating effect. However, as the electron energy increases, the collision cross-section between the electron and other particles gradually increases, resulting in a greater loss of electron energy. In general, in one cycle of the applied voltage, the energy gained by the electrons is greater than the energy lost by the collision. Therefore, the amplitude of the electron energy gradually increases. At the same time, as the frequency of the applied voltage increases, the applied voltage in the cycle increases correspondingly, resulting in a greater amplitude of electron energy. In other words, at the same time as when the frequency of the applied voltage increases, the amplitude of the electron temperature gradually increases.

### 4.3. Surface Charge Accumulation Characteristics

**Figure 10:**
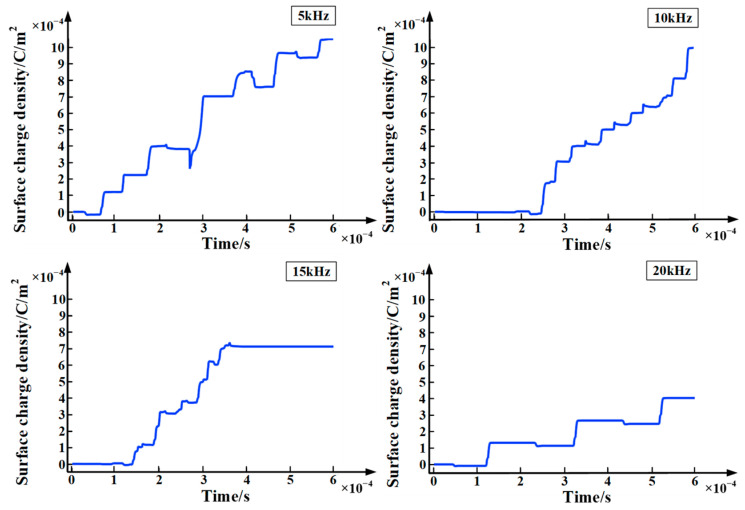
Chart of surface charge density with time at different frequencies.

During the partial discharge, there are a large number of charged particles in the plasma region. Under the influence of the strong vertical component of the electric field, these particles gradually move to point A, causing charge accumulation. The surface charge accumulated will store some electrostatic energy around point A. Usually, these electrostatic energies are stable. However, when the energy reaches a critical state, the balance is broken and the energy stored by the surface charge will be released. These energies can promote the collision, ionization, and recombination of charged particles, and affect the development of partial discharge. Figure 10 shows the chart of surface charge density with time at different frequencies. It can be seen from the figure that a large amount of positive charge accumulates near point A, which is basically consistent with the conclusions found in the literature [22]. In addition, due to the existence of a large number of charged particles, the surface charge density increases significantly with the extension of the applied voltage time. Furthermore, as the frequency of the applied voltage increases, the surface charge density gradually decreases. When the applied voltage is in the positive polarity, the electrons and positive ions generated by the collision ionization near point A, respectively, move to the plate electrode at point A and metal particles under the force of the electric field. Since the electric field intensity at point A is relatively high, electrons can easily reach the plate electrodes. However, positive ions will be blocked by the epoxy interface during migration and form a surface charge accumulation. When the applied voltage is in the negative polarity, the positive ions and electrons generated by the impact ionization, respectively, move to the plate electrode, and the metal particles under the force of the electric field. Under a weak electric field, electrons easily react with neutral particles to become negative ions in the process of moving. On the one hand, the presence of positive ions in the ionization zone will restrict electrons to move outward; on the other hand, negative ions will partake in a neutralization reaction with positive ions. The effects of these two aspects lead to relatively little accumulation of negative charges at the epoxy interface. Therefore, in each cycle of the applied voltage, a large amount of positive charge accumulates near point A. Furthermore, as the frequency of the applied voltage increases, the time of each half-cycle gradually decreases. Due to the lack of a continuous electric field, the accumulation of surface charges gradually decreases. In other words, as the frequency increases, the surface charge density gradually decreases.

From a microscopic point of view, as the frequency of the applied voltage increases, the amplitude of the electron temperature shows an increasing trend, while the surface charge density shows a downward trend as the frequency increases. These two factors make partial discharge severest when the frequency of applied voltage is 15 kHz. This is consistent with the factors considered for the severity of partial discharges (surface electric field distortion, surface charge accumulation).

## 5. Epoxy Interface Particle Defect Partial Discharge Experiment

**Figure 11:**
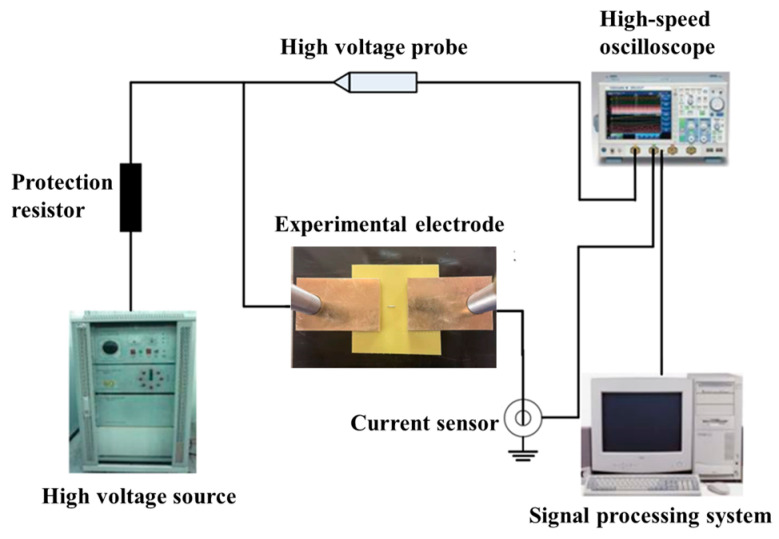
The experimental platform for partial discharge of epoxy interface particle defects under high-frequency sinusoidal voltage excitation.

For this paper, an experimental platform for the partial discharge of epoxy interface particle defects under high-frequency sinusoidal voltage excitation was built, as shown in Figure 11. The experimental platform included a high-frequency sinusoidal voltage source, protection resistor, high-voltage probe, high-frequency partial discharge measurement system, and an experimental electrode. Among them, the high-frequency partial discharge measurement system consisted of a high-speed digital oscilloscope (YOKOGAWA DL6154), a pulse current sensor (ETS-Lindgren 93686-8 type), and a signal processing system. The sensitivity of the pulse current sensor was 1 mA/0.05 mV, and the measurement bandwidth was 10 kHz~100 MHz. The signal processing system was self-developed, and the characteristic parameters of the partial discharge signal at high-frequency were obtained by wavelet decomposition and noise reduction processing of the current sensor signal.

The experimental electrode was made of brass, and the epoxy resin was 100 mm in diameter, with a 2 mm thickness circular specimen cast being provided by Shandong Taikai Transformer Co. Before the experiment, the sample was cut into 40 mm × 40 mm, and the surface of the sample was wiped with anhydrous ethanol and dried at a low temperature in a room temperature drying oven. The epoxy resin plate was fixed to the experimental plate by screws to ensure that the electrodes were in close contact with the epoxy resin plate. The electrode spacing was 10 mm, and the metal particles were aluminum particles with a length of 4 mm, the end of which was 2 mm from the high-voltage electrode and 4 mm from the ground electrode. Approximately 10 kV high-frequency sinusoidal voltage of different frequencies was applied to the experimental electrode to conduct partial discharge experiments on the epoxy interface particle defects.

Based on the aforementioned partial discharge experimental platform, the partial discharge characteristics of metal particles attached to the surface at different frequencies and the degradation law of epoxy resin surface insulation properties were studied.

### 5.1. Partial Discharge Experimental Research

**Figure 12:**
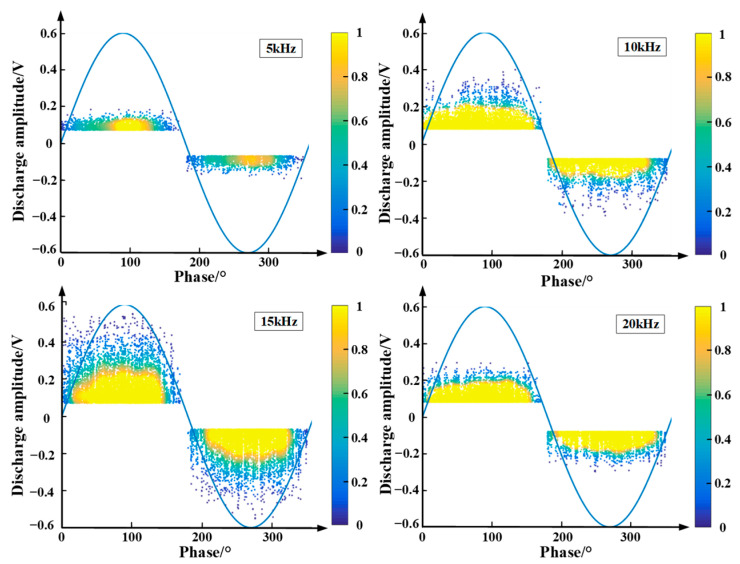
PRPD chart of partial discharge of epoxy interface particle defect at different frequencies.

The Phase Resolved Partial Discharge (PRPD) at different frequencies is shown in Figure 12. The denseness of the dots in the figure reflects the discharge density, and the yellow area represents the discharge dense area [23]. As can be seen from the figure, the partial discharge occurs throughout the time, and is most intense near the peak of the sinusoidal voltage, while its discharge voltage amplitude is larger near the peak. When the applied voltage frequency is 15 kHz, the discharge density is significantly higher than other frequencies, and the discharge voltage amplitude is the largest, so it can be considered that the partial discharge is the most serious at the frequency of 15 kHz. This is consistent with the simulation results, which fully verifies the reasonableness of the simulation model and the accuracy of the calculation results.

### 5.2. Overall Morphological Analysis of Surface Damage

**Figure 13:**
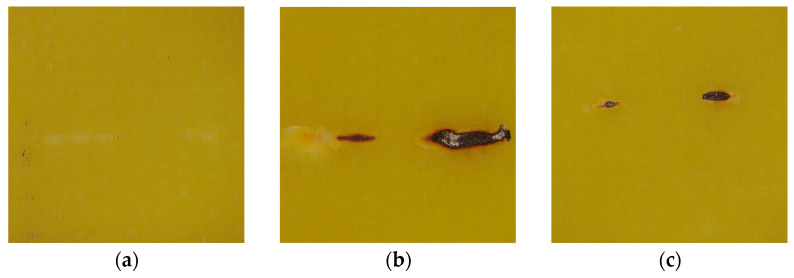
Overall shape of the damaged area. (**a**) 10 kHz; (**b**) 15 kHz; (**c**) 20 kHz.

The epoxy resin samples of the same size were selected and sinusoidal voltages with frequencies of 10 kHz, 15 kHz, and 20 kHz were applied to them, all of which had an amplitude of 10 kV. The carbon traces generated after partial discharge on the epoxy resin surface are shown in Figure 13 (below).

The 15 kHz and 20 kHz samples have obvious carbon marks, with the carbon marks at 15 kHz being significantly darker and increasing in width and length. At 10 kHz, only the ends of the particles show degradation points, and the ends of the particles show shallow damage marks.

### 5.3. Surface Topography Analysis Based on SEM Measurements

**Figure 14:**
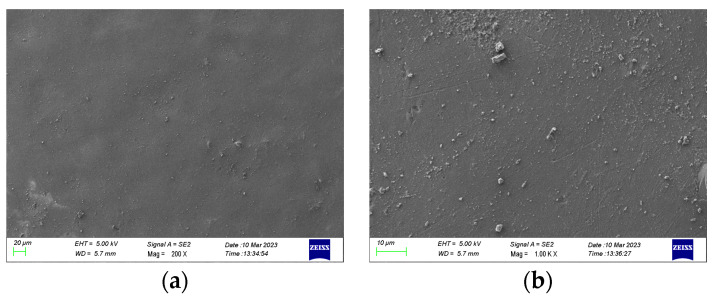
Epoxy surface topography without discharge based on SEM measurements. (**a**) 200×; (**b**) 1000×.

Scanning electron microscopy (SEM) can observe the microscopic morphology of the material surface. The SEM was used to observe the original specimens at the magnification of 200× and 1000×, and the observation results are shown in Figure 14. It can be seen from the figure that the surface of the epoxy resin without deterioration is relatively flat, and only a small amount of impurity particles is attached to the surface.

**Figure 15:**
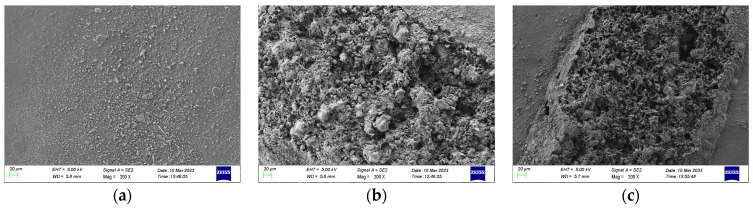
Epoxy surface topography near high-voltage electrodes based on SEM measurements. (**a**) 10 kHz; (**b**) 15 kHz; (**c**) 20 kHz.

**Figure 16:**
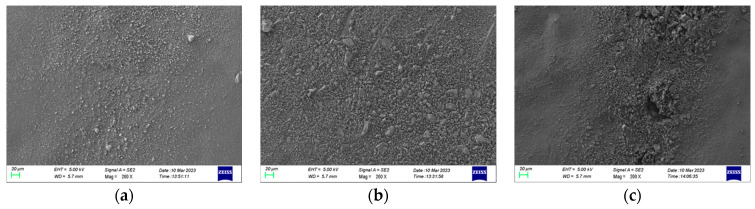
Epoxy surface topography near ground electrodes based on SEM measurements. (**a**) 10 kHz; (**b**) 15 kHz; (**c**) 20 kHz.

SEM was used to observe the damage area of the specimen near the high-voltage electrode side and the ground electrode side, respectively, at 200 times magnification, and the observation results are shown in Figure 15 and Figure 16.

As can be seen from the figure, the damage is more severe in the area near the high-voltage electrode compared to the ground electrode side. In the area near the high-voltage electrode, many particle-like bumps are produced at 10 kHz. At 15 kHz and 20 kHz, the surface microscopic morphology is complex, with a large number of particles, holes, and lamellar structures of a large diameter, which may be the result of multiple partial discharges. In the damage area near the ground electrode, the main damage on the epoxy surface is a large number of particulate bumps. At 15 kHz and 20 kHz, specimens produce a larger diameter and more dense distribution of particles, and the degree of fragmentation is more severe at 15 kHz.

### 5.4. Surface Topography Analysis Based on AFM Measurements

**Figure 17:**
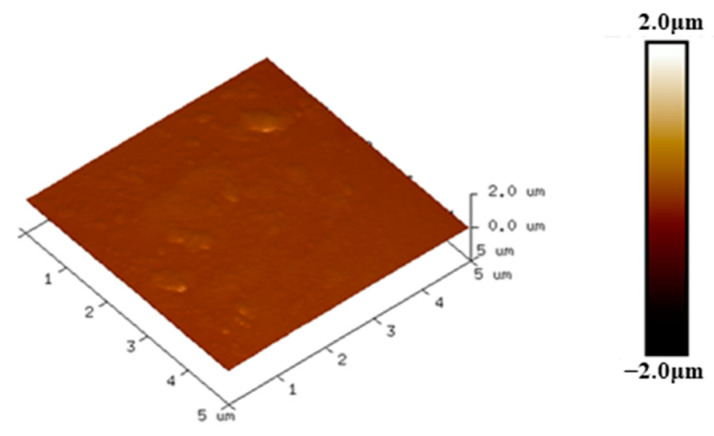
Epoxy surface topography without discharge based on AFM measurements.

Atomic force microscopy (AFM) can observe more in-depth microscopic details on the material surface than SEM. In this paper, the AFM test results of damaged samples at different frequencies were analyzed by using Nanoscope Analysis software. The scan range was set to 5 × 5 μm, and the vertical coordinates were unified from −2 μm to 2 μm. The analysis results are shown in Figure 17.

**Figure 18:**
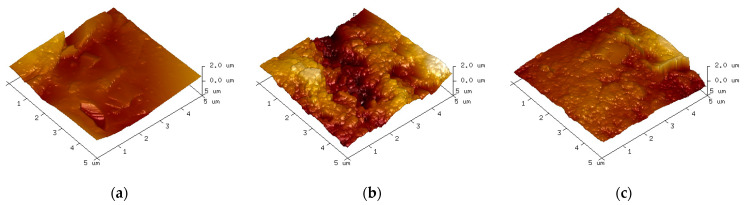
Epoxy surface topography near high-voltage electrodes based on AFM measurements. (**a**) 10 kHz; (**b**) 15 kHz; (**c**) 20 kHz.

**Figure 19:**
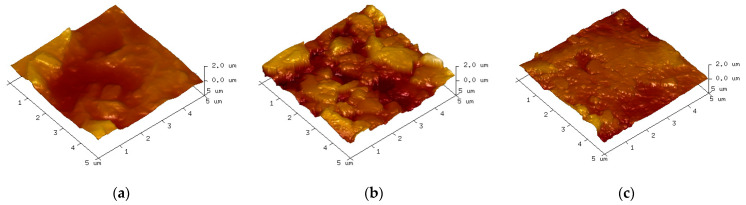
Epoxy surface topography near ground electrodes based on AFM measurements. (**a**) 10 kHz; (**b**) 15 kHz; (**c**) 20 kHz.

Figure 17 corresponds to the undamaged epoxy resin, which has a relatively flat surface and less roughness. Figure 18 and Figure 19 show the AFM test results in the area near the high-voltage electrode and the ground electrode, respectively. Figure 18a shows the analysis results of the damaged specimen near the high-voltage electrode at 10 kHz, where the surface has some protrusions, but the overall surface is still flat. The samples at 15 kHz and 20 kHz have more protrusions on the surface, and the surface morphology changes significantly. However, at 15 kHz, the surface of the sample has more protrusions and is more densely distributed, producing more obvious grooves and greater surface roughness. In the area near the ground electrode, the epoxy surface is significantly less damaged than the area near the high-voltage electrode. Compared to other voltage frequencies, the surface of the specimens at 15 kHz shows large protrusions and the most severe degradation.

The degree of surface damage reflects the severity of partial discharge [24]. As mentioned before, the surface damage of the epoxy samples was most severe at 15 kHz. Additionally, the damage in the epoxy region near the high-voltage electrode was more severe at the same frequency. This is consistent with the results of the simulation in the previous paper. Therefore, it can be concluded that the partial discharge of the epoxy resin with metal particles on the surface is also the most severe when the applied voltage frequency is 15 kHz.

## 6. Conclusions

This paper proposes a partial discharge detection method for the particle defects of insulators under high-frequency sinusoidal voltage excitation. For epoxy insulators attached with metal particles, a two-dimensional plasma simulation model for partial discharge of epoxy interface particle defects under a plate–plate electrode structure was built to realize the dynamic simulation of the development process of partial discharge. By studying the microscopic mechanism of partial discharge, the spatial and temporal distribution characteristics of microscopic parameters such as electron density, electron temperature, and surface charge density are obtained. The accuracy of the model is verified from two aspects of surface damages and discharge intensity through experimental means. Based on the partial discharge plasma simulation model of epoxy interface particle defects built in this paper, the following conclusions can be drawn:

(1) The partial discharge is mainly concentrated at three junctions of the electrode: the epoxy interface and the air (A, C) and the end of the metal particles near the electrode side (B). Furthermore, in view of the amplitude and distribution range of electron density, the electron density of point A is far higher than that of point C, and the electron density of point C is far higher than that of point B.
(2) Surface damage occurs in the epoxy region at both ends of the metal particles, with the damage in the region near the high-voltage electrode being stronger than that in the ground electrode region. When the frequency of the externally applied voltage is 15 kHz, the degree of damage to the epoxy surface is significantly higher than other voltages.
(3) Through experiments and simulation analysis, it is clear that the partial discharge of epoxy interface particle defects is severest when the frequency of the applied voltage is 15 kHz. The reasons for this are as follows: as the frequency of the applied voltage increases, the amplitude of the electron temperature shows an increasing trend, while the surface charge density shows a downward trend as the frequency increases. These two factors mean the partial discharge severest when the frequency of applied voltage is 15 KHz.

In this paper, the microscopic mechanism is analyzed by focusing on the partial discharge process of metal particles at epoxy interfaces under high-frequency excitation. Further, the effect of frequency on partial discharge is clarified, and an effective detection method is provided for the detection of epoxy insulator particulate defects in GIL.

## Figures and Tables

**Table 1:** Plasma chemical reaction during partial discharge.

Number	Reaction Equation	Energy Loss	Reaction Rate Coefficient
1	e + A ⇒ p + 2e	15 eV	-
2	e + A ⇒ n	-	-
3	e + 2A ⇒ n + A	-	2 × 10^−41^ × (300/T_e_) × NA^2^
4	e + p ⇒ A	-	5 × 10^−14^
5	n + p ⇒ 2A	-	5 × 10^−12^

In the table, “-” means that this parameter does not need to be set separately. The reaction rate coefficient of ionization reaction and attachment reaction can be replaced by Townsend coefficient; NA is Avogadro constant.

## Data Availability

The original data from the experiment are not publicly available and can be obtained upon reasonable request by contacting the corresponding authors.
